# Entrained Metal Aerosol Emissions from Air-Fired Biomass and Coal Combustion for Carbon Capture Applications

**DOI:** 10.3390/ma11101819

**Published:** 2018-09-25

**Authors:** Karen N. Finney, János Szuhánszki, Leilani I. Darvell, Benjamin Dooley, Kris Milkowski, Jenny M. Jones, Mohamed Pourkashanian

**Affiliations:** 1 Energy 2050, Department of Mechanical Engineering, University of Sheffield, Sheffield S3 7RD, UK; j.szuhanszki@sheffield.ac.uk (J.S.); k.milkowski@sheffield.ac.uk (K.M.); m.pourkashanian@sheffield.ac.uk (M.P.); 2 School of Chemical and Process Engineering, Faculty of Engineering, University of Leeds, Leeds LS2 9JT, UK; l.i.darvell@leeds.ac.uk (L.I.D.); pm07bd@leeds.ac.uk (B.D.); j.m.jones@leeds.ac.uk (J.M.J.)

**Keywords:** biomass, bioenergy with carbon capture and storage (BECCS), metal aerosol emissions, alkali metals, transition metals

## Abstract

Biomass energy with CO_2_ capture could achieve net negative emissions, vital for meeting carbon budgets and emission targets. However, biomass often has significant quantities of light metals/inorganics that cause issues for boiler operation and downstream processes; including deposition, corrosion, and solvent degradation. This study investigated the pilot-scale combustion of a typical biomass used for power generation (white wood) and assessed the variations in metal aerosol release compared to bituminous coal. Using inductively coupled plasma optical emission spectrometry, it was found that K aerosol levels were significantly greater for biomass than coal, on average 6.5 times, with peaks up to 10 times higher; deposition could thus be more problematic, although Na emissions were only 20% of those for coal. Transition metals were notably less prevalent in the biomass flue gas; with Fe and V release in particular much lower (3–4% of those for coal). Solvent degradation may therefore be less severe for biomass-generated flue gases. Furthermore, aerosol emissions of toxic/heavy metals (As/Cd/Hg) were absent from biomass combustion, with As/Cd also not detected in the coal flue gas. Negligible Cr aerosol concentrations were found for both. Overall, except for K, metal aerosol release from biomass combustion was considerably reduced compared to coal.

## 1. Introduction

Dealing with the key environmental concern of anthropogenically-induced climate change is imperative, but providing cost-effective, reliable energy for the growing global population and their increasing demands are also essential. Whilst there is a necessity to ‘keep the lights on’, we need to ensure that this is not done at the expense of the environment; thus energy generation needs to be both sustainable and efficient. Consequently, stringent emissions targets are now in place to limit global temperature rises to 2 °C, with a particular focus on CO_2_ reductions from carbon intensive sectors, such as power generation.

### 1.1. Lowering CO_2_ through Net Negative Emissions

Using fuels with a lower carbon intensity, such as biomass, can reduce net CO_2_ emissions from power generation that currently focuses on fossil fuels. Carbon capture and storage (CCS) can also offer long-term options for low-carbon thermal power stations, with post-combustion capture extensively researched. Whilst there is now a wealth of knowledge for coal-fired CCS (including commercial-scale demonstration projects [1,2]), there is a distinct lack of operational experience when applied in the context of biomass. Thus far, only limited experimental work has been conducted on the assessment of integrating biomass with CCS technologies. Dedicated biomass energy with CCS (BECCS) has the potential to achieve net negative emissions—a carbon-negative solution that permanently removes atmospheric CO_2_—which can be strategically important for meeting carbon budgets [3]. BECCS is just one of a limited number of technologies that can achieve net negative emissions and therefore has been heralded as a key CO_2_ mitigation strategy [4,5].

### 1.2. The Challenges of Biomass and BECCS

Even though BECCS has the potential to provide a range of benefits, there remain a number of technology barriers that need to be better understood and overcome prior to commercial-scale deployment. The properties and composition of biomass differ somewhat to other solid fuels, most notably their metal and inorganic content [6,7,8]. These can impact on combustion and downstream processes, including heat recovery, environmental control, and CO_2_ capture. Dealing with the differences in feedstock characteristics is a key challenge, not only to biomass use in dedicated or co-fired plants, but also to integrated BECCS systems.

Alkali metals cause deposition within the boiler through slagging (high-temperature regions) and fouling (lower-temperature areas), due to the formation of salts and silicates with relatively low melting points [7,8,9,10]. This can detrimentally impact heat flux/transfer as they build up over time and limit efficiency, necessitating regular maintenance [8,11]. Other elements can also contribute to slagging, such as oxides of Si, Al, and Fe [12]. Furthermore, acidic elements (N/S/Cl) can form inorganic anion compounds, which react with the metal oxides and attack the surfaces under the deposits, causing high temperature corrosion of the tube bundles [8,10]. Light alkali metals (K, and to a lesser extent Na) and acidic species tend to be more prevalent in biomass than coal (more so for herbaceous rather than woody varieties), thus the phenomena are exacerbated for biomass-fired plants due to their presence as oxides, sulfates, chlorides, and phosphates [6,13,14,15,16].

Furthermore, lighter, volatile metals (alkali, transition, and heavy metals, e.g., Cu/Cr/Ni/Fe/V) can aid oxidative solvent degradation in post-combustion capture plants if these are carried over [17,18,19]. They can dissolve and catalyze oxidation, even at low concentrations, or take part directly in reactions—with both pathways affecting a range of alkanolamine solvents [19,20,21,22]. Volatile metals, present as strong anions have the potential to react with amine cations to form heat-stable salts, destroying the solvent. Transition (and heavy) metals tend to be concentrated in the flyash (rather than the coarser, bottom ash) [23] and submicron particulates (ultrafine or nanoparticles) can bypass the solid-phase separation devices to be carried over to the capture plant, where they build up. Moreover, biomass ash demonstrates much higher enrichment factors than coal ash for a number of metals, including Cu and Cr [24]. Oxidative degradation is initiated by the presence of free radicals and thus the inclusion of redox reactive metal ions, in solid or gaseous (aerosol) form, may generate significant amounts of these, accelerating degradation rates [22]. These can also cause corrosion in the system. Additionally, these metals have the potential to catalyze other reactions in the high-purity CO_2_ stream. Although much is known about the ill effects that metal emissions can have, there is scant empirical data concerning their measurement from real plants.

### 1.3. Trace Element Emissions Monitoring Challenges

Whilst gaseous emissions from combustion have been extensively investigated, there is limited research into the quantification of trace element emissions from such processes. The few studies that have been conducted largely focus on coal and wastes rather than dedicated biomass, with much of the reporting and monitoring focusing on toxic metals, mainly mercury, which is of interest for health and environmental reasons [25,26,27,28,29,30]. More recently, studies are emerging that explore the release of inorganics from biomass combustion, including K, Na, Zn, and Pb emissions from a range of feedstocks, such as miscanthus, spruce, agricultural residues, waste wood, and straw [31,32,33,34,35].

The operational variables of the furnace, mainly the combustion temperature and O_2_ levels, have been found to be key influencing parameters, playing significant roles in determining the volatilization of metals, where increasing furnace temperatures increased the release ratios of volatile and semi-volatile ash-forming elements [27,28,33,34]; K release in particular increases with combustion temperatures [31,32,33]. Gas-phase species, particularly of non-volatile elements, can be impacted by the Cl and Ca content, and emissions of K were found to decrease with increasing silicate content in the fuel [31,32]. It is thought that the release mechanisms for Na are similar to those for K [33,34]. Ash interactions could also limit the vaporization of specific elements, such as As, which may dissolve in silicate glass or form calcium-based compounds [36]. Certain semi-volatile toxic metals (As/Cd) can volatilize and be emitted in the gas-phase near the combustion zone, with flame temperature being a deciding factor; subsequent heterogeneous transformation can take place further downstream, where submicron particles nucleate/coagulate via surface reactions, changing the form in which these species are present in the flue gas [37,38].

### 1.4. Aims and Objectives

The primary aim of this research was to assess the variations in species and concentrations of entrained metal aerosols in the flue gases for different fuels under air-fired conditions—comparing coal and biomass. This was achieved through pilot-scale combustion trails with an inductively coupled plasma optical emission spectrometer (ICP-OES) to monitor emissions of metals that are toxic and/or easily vaporized and/or cause operational issues.

## 2. Materials and Methods

The UK’s National Pilot-scale Advanced CO_2_ Capture Technology (PACT) Core Facilities were utilized for this research; they include a pulverized fuel combustor for coal and biomass combustion, and an extensive emissions analysis suite, amongst a variety of other advanced combustion and carbon capture facilities.

### 2.1. Fuel Feedstocks

Two fuels were used here, both of which are typical for power generation in thermal power plants. The fossil fuel was a bituminous Columbian coal, from the El Cerrejon region, whilst the biomass was North American Grade A white wood pellets manufactured from forestry residues. Each fuel was pulverized before firing in the combustion test facility (CTF) considered in the next section.

The ultimate and proximate analyses are provided in Table 1 for the coal and biomass, along with their major and trace element contents. The proximate analyses were performed in duplicate to the following British Standards: BS EN 14774-3:2009 (moisture), BS EN 15148:2009 (volatiles), and BS EN 14775:2009 (ash) for the biomass; and BS ISO 11722:2013 (moisture), BS ISO 562:2010 (volatiles), and BS ISO 1171:2010 (ash) for the coal, as described in Dooley [39]. The elemental composition was determined in duplicate using a CE Flash EA 1112 Series analyzer (Thermo Quest CE Instruments, Italy) [39]. The major components and trace metals of the ash were subcontracted to an accredited analytical laboratory.

### 2.2. PACT Combustion Test Facility

**Figure 1:**
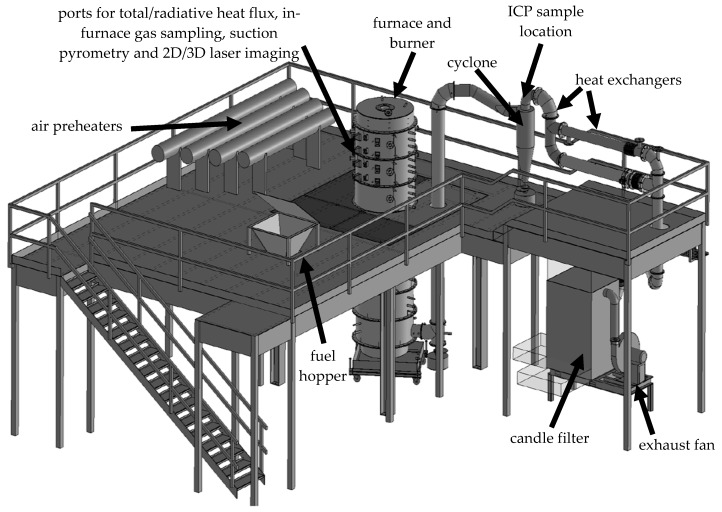
Schematic of the combustion test facility (CTF), identifying the main components and inductively coupled plasma (ICP) sampling location.

PACT houses a down-fired, cylindrical furnace, with interchangeable burners and feeding systems for coal and biomass combustion; a schematic of the main components is shown in Figure 1. This CTF is a pulverized fuel reactor, 0.9 m in diameter and 4 m in height that can be operated in air- or oxy-fired mode; when used under air-firing conditions, it can be coupled with the on-site, solvent-based post-combustion carbon capture plant for CCS/BECCS applications.

The primary air carries the pulverized fuel into the burner at the top of the furnace chamber—a scaled version of a commercial Doosan Mark III, low-NOx burner was used for coal firing and a General Electric burner designed for biomass firing was used for the wood. Swirled secondary and tertiary air provide the rest of the oxidizer and are preheated to 250 °C. Table 2 outlines the settings and parameters used for the operation of the furnace herein.

### 2.3. Metal Aerosol Emissions Analysis

Entrained metal aerosols were measured in the flue gas from the CTF, at the location outlined in Figure 1 (cyclone exit), using continuous online ICP-OES, a state-of-the-art diagnostics tool available at PACT. The Spectro CIROS^CCD^, containing a CIRcular Optical System with Charge Coupled Device optics, quantitatively detects multiple elements simultaneously by ionizing atoms with electrical plasma. The emission spectra are formed by inductively coupling radiofrequency energy in the argon gas plasma.

#### 2.3.1. System Description and Specification

**Figure 2:**
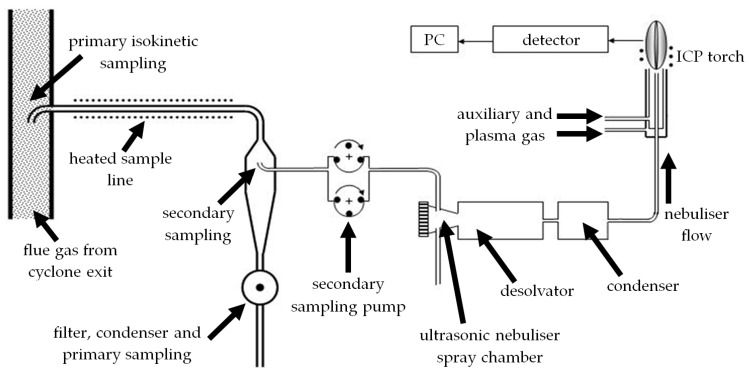
Sampling system for the ICP and the sample introduction pathway to the torch.

The instrument herein can assess the spectral lines of a large number of elements, including volatile and non-volatile species, down to ultratrace levels. It has a solid-state detector-based ICP with continuous 1st Order wavelength coverage. Its optical system merges two high resolution Paschen-Runge polychromators into a single mount; the demountable torch was custom-built by Spectro, Kleve, Germany, with a radial view configuration and operates at ~6000 K. It has an injector i.d. of 1.8 mm, which allows for the higher power and coolant flowrate required to sustain the plasma under the atmospheric conditions used (Table 3). The primary flue gas sampling from the CTF was isokinetic, with the secondary sample location within the main 40 m heated line, taken via a peristaltic pump, as detailed in Figure 2, at a sample flowrate of 200 mL/min (Table 3). The heated lines in the sampling system were operated at 180 °C to minimize line losses and eliminate moisture condensation, with the temperature at the primary sampling point also monitored. The desolvator and condenser were used as outlined in Figure 2 to rapidly condense out the moisture in a controlled manner, ensuring a dry aerosol sample was provided to the ICP torch.

#### 2.3.2. Element Detection and Calibration

The ICP was used to determine the variations in metal aerosol concentrations in the flue gases from coal and biomass combustion. Based on the key elements found in the fuel analysis, calibrations were performed to the detection limits shown in Table 4. Different standards were used at a variety of concentrations consistent with the likely levels to be observed in the flue gases. The stock solutions included a multi-element standard, plus a separate standard for Hg. All calibrations were performed at appropriate levels of CO_2_ in the incoming flow −14.9 (±2%) vol%, matching the exhaust gas compositions of between 14.6 and 16.1 vol%—as this has been previously shown to notably effect instrument calibration and the species detection limits by altering signal sensitivity, with different impacts observed for different elements [27,29]. Due to the variations in the exhaust gas composition (namely CO_2_ content), as well as human errors introduced during calibrations, measurement accuracy may be impeded slightly, however this will not affect the ratios calculated.

## 3. Results

**Figure 3:**
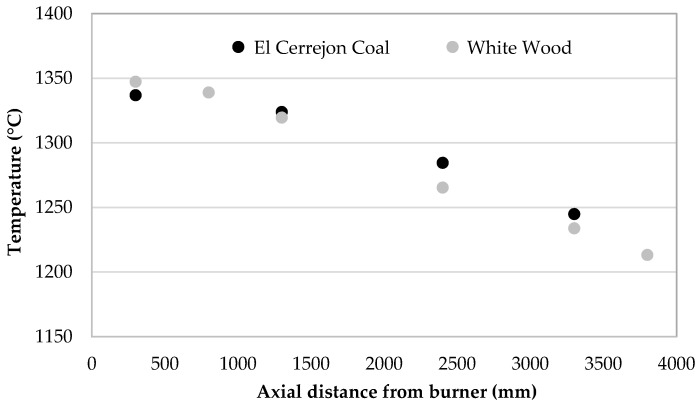
Furnace temperatures for coal- and biomass-fired cases, recorded with fixed thermocouples at 250 mm radial distance from furnace axis and various axial distances from the burner.

The furnace temperatures were recorded for both the coal- and biomass-fired cases using ceramic sheathed thermocouples fixed at various axial distances from the burner exit. All thermocouples were fixed at 250 mm from the inner wall and 200 mm from the furnace center line. Both cases were fired at the target thermal rating of 200 kW (Table 2), and so produced similar temperature trends along the height of the furnace, with very similar exit temperature profiles—at 3300 mm from the burner, the temperature difference between the two cases was 11 °C. Although flame temperatures within the sheer mixing zone may differ more significantly, furnace temperature trends produce very similar profiles, with the maximum difference recorded as 19 °C at 2400 mm axial distance from the burner outlet, as shown in Figure 3. This indicates that that particles of both coal and biomass cases have travelled through similar temperature zones along the furnace.

**Figure 4:**
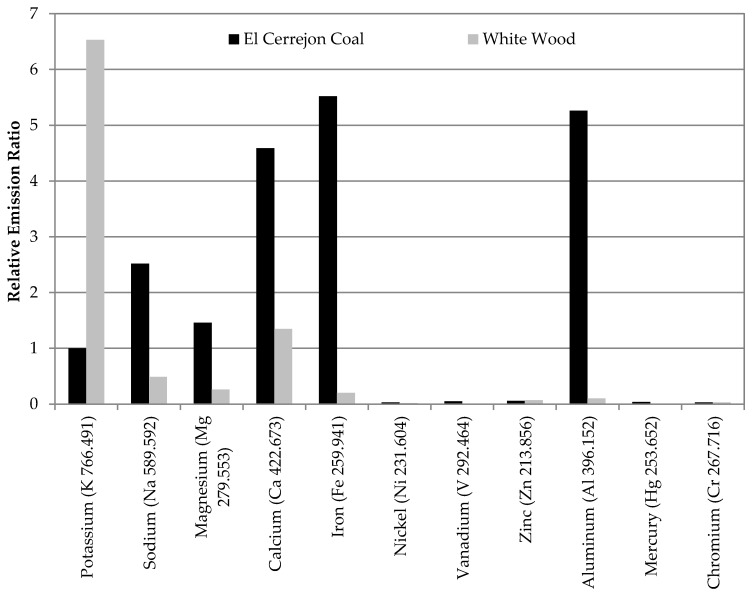
Average relative emission concentration ratios from coal and biomass combustion, compared to the average data for potassium from coal combustion (standardized to 1).

Metal aerosol emissions were monitored from the CTF, comparing the species and relative concentrations of a range of elements for the coal and biomass test campaigns, based on the element-specific spectra detailed in Table 4. Table 5 compares the ratios of each element relative to the average data achieved for coal for that species, with Figure 4 outlining the relative ratios for each in comparison to the average data for potassium from coal combustion. Although Cu, As, and Cd spectral lines were calibrated at low concentrations, emissions as aerosols were not detected and are therefore not reported in Table 5 or Figure 4. The sections below discuss the alkali/alkali earth, transition, and heavy/toxic metal emissions observed for the different fuels in turn.

### 3.1. Alkali and Alkali Earth Metals

#### 3.1.1. Potassium and Sodium

**Figure 5:**
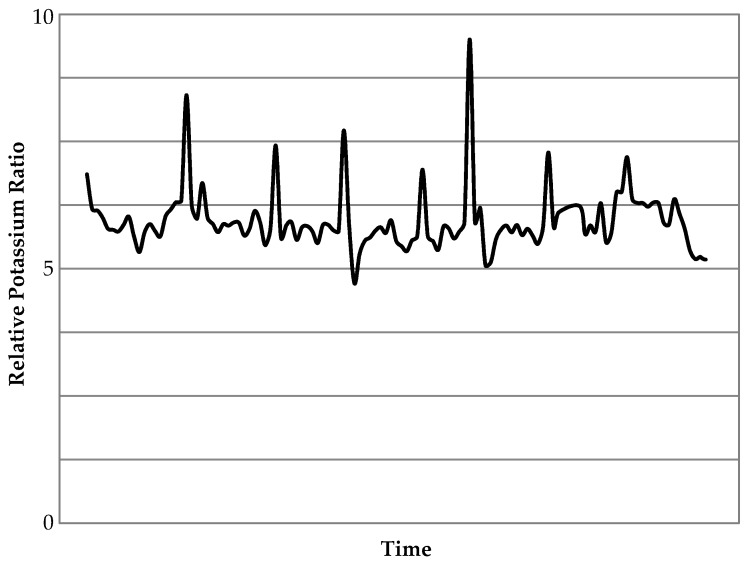
Real-time emission concentration ratios of K from biomass combustion, relative to the data standardized for K for the coal average; time period shown is 1 h.

Elevated levels of K were found in the biomass feedstock, although there was still a notable concentration in the coal sample (Table 1). Consequently, aerosol emissions of K, which is quite a volatile element, were much higher in the biomass flue gas than that from the coal. The combustion of the wood resulted in average K emissions that were ~6.5 times greater than those for coal (Figure 4), with peaks of up to 10 times (Table 5). This is the only element that was found in higher concentrations as an aerosol in the flue gas from white wood combustion compared to the coal. Figure 5 outlines real-time data recorded for one of the biomass combustion tests; this shows a range of different peaks, some of which are around 9 or 10 times greater than those for coal. These peaks lasted for relatively short periods of time and are likely to be due to natural variations in the fuel composition and instabilities in the flame.

Na was present in both fuels, but the coal flue gases demonstrated greater concentrations of aerosols, with peak ~1.6 times higher than the average. The sodium concentration in the biomass flue gas by contrast was significantly lower—just 20% of that from coal; with peak levels a third of the coal average. The biomass generated high levels of potassium aerosols, whilst the sodium aerosols produced from coal combustion were greater than for potassium; thus there is potential for issues caused by deposition from both fuels.

#### 3.1.2. Magnesium and Calcium

Both these metals were found in high concentrations in the initial fuels, with high levels of Ca in particular in the biomass; magnesium was also more prevalent in the wood. Whilst these were found as aerosols in the exhaust from both fuels, they were much more prevalent for coal. The maximum levels observed in the coal flue gases were around twice the average for the overall tests. Average Mg aerosol concentrations for biomass were ~20% of those from the coal, with peak emissions also much lower. For Ca, high aerosol concentrations were detected for the coal, with the average and peak levels for biomass around a third of those for the coal. Ca emissions from coal were amongst the highest levels recorded (Figure 4).

### 3.2. Transition Metals

#### 3.2.1. Copper

Although Cu was found in relatively small concentrations in both fuels, taking into account the relatively high melting points of both its oxide and sulfide compounds, it was not expected that this element would be particularly dominant as an aerosol. Cu was not detected in either of the flue gas samples, despite the low detection limit (Table 4).

#### 3.2.2. Iron

There was a considerable amount of Fe found in the coal feedstock, with much lower levels in the biomass fuel. As a result, there were significant levels of Fe aerosols seen in the coal flue gas samples—in fact, the highest concentrations for all the metals monitored for coal in this study (Figure 4). Although high average concentrations were noted for coal combustion, peak levels were considerably greater, as detailed in Table 5. The differences in Fe detected between the coal and the biomass show that the iron present in the two fuels is likely to be found in different compounds, which impacts on their volatility and thus their partitioning and release as aerosols. Fe aerosols were relatively low from the white wood combustion by comparison. For both fuels, Fe was the most dominant transition metal aerosol found to be present (Figure 4).

#### 3.2.3. Nickel

Ni, although found in low levels for both fuels, was seen in higher concentrations in the coal. Relatively low aerosol concentrations were detected in the flue gases though; average readings for the biomass tests were ~70% of those for coal, with peak readings of Ni aerosols for the biomass equivalent to the average data acquired for the coal. Ni aerosol levels were small compared to those recorded for other elements (Figure 4).

#### 3.2.4. Vanadium

V was also found in higher concentrations in the coal fuel (the most dominant trace metal), with low levels of V in the wood. Consequently, vanadium aerosols were more prominent in the coal flue gas, with peak emissions twice the average; overall concentrations however remained low, as seen from Figure 4. By comparison, V emissions from the biomass tests were minimal, with peak emissions only 5% of the coal flue gas average.

#### 3.2.5. Zinc

Of all the trace metal species, Zn was the most predominant in the biomass fuel, with concentrations of more than 10 mg/kg (Table 1). Levels of zinc aerosols were found to be relatively similar between the coal and biomass combustion flue gases. The average concentrations of Zn aerosols were higher for the biomass combustion (the only other element apart from potassium which showed higher aerosol levels), although emission peaks were greater for the coal. In contrast to other species though, such as the alkali/alkali earth metals and iron, Zn aerosols generation was low from both fuels.

### 3.3. Other Metal Species (Including Toxic/Heavy Metals)

#### 3.3.1. Aluminum

Al was found to be quite prominent in the coal feedstock and was also detected at notable levels in the wood, therefore aerosol emissions monitoring for this element was undertaken. Al aerosol emissions were in fact amongst the highest seen for the coal combustion—second only to iron—with peak levels around twice those of the average. Emissions of Al aerosols from the combustion of the white wood by comparison were minimal, as seen from Figure 4, and were broadly similar to the levels of transition metal aerosols from biomass considered in Section 3.2.

#### 3.3.2. Mercury

Hg was found in only negligible amounts in both the fuels used here, as for the other heavy/toxic metals. However, as it is a highly volatile element, it was thought that the partitioning would show this to be found in the vapor phase, detectable with the ICP. Hg was not detected at all in the biomass flue gas, as shown in Table 5, though low levels were generated from coal combustion. As seen from Figure 4, Hg aerosol levels were very small compared to the other elements considered herein. Peak concentrations for the coal flue gas were around 1.7 times that of the average reading, but were all above the lower detection limit (Table 4).

#### 3.3.3. Chromium

Cr was found in the initial fuels and was more prevalent in both than Cd. Chromium aerosols were detected in similar concentrations for both coal and biomass combustion, and although these were largely below the instrument detection limit, they were noted as present. Average concentrations were similar, although the coal tests had higher peak concentrations; the maximum Cr aerosol concentration for the biomass tests was around 1.5 times greater than that of the average for the coal. Compared to the other metals considered here, the toxic and heavy elements, such as Cr, were found only in limited aerosol concentrations for both fuels.

#### 3.3.4 Cadmium

Cd was in fact not detected in the flue gases of either of the fuels during the combustion tests, despite the low detection limit.

## 4. Discussion

Aerosol emissions of a variety of metal species were found to be present in both the coal and biomass flue gases, as detected via real-time ICP analysis. The elements present as aerosols, however, varied considerably in their concentrations between the two fuels. For the biomass, the highest concentration was for potassium, with levels 6–10 times greater than the average data for coal. As reported, similar differences in K concentrations were found in the initial feedstocks. It was noted in Section 1.3 that emissions of K decrease with increasing silicate content, and thus potassium release from the coal may have been impeded by the high SiO_2_ content of the coal ash (Table 1). Mason et al. [40,41] investigated the fate of potassium during the combustion of single biomass particles, observing temporal release patterns of gas-phase K and finding a strong correlation between the K content of the fuel and the release rate, especially the peak. Tightly controlling combustion, especially temperature profiles, can optimize burnout and aid in controlling K release and its eventual fate (partitioning).

With the exception of K, it was only Ca that was found in particularly elevated concentrations in the biomass flue gas, although this was still lower than for coal. In fact, it was only potassium aerosols that were found in higher concentrations in the flue gases from biomass combustion compared to that of the coal (Figure 4). Coal, by comparison, had a number of species with high aerosol concentrations, especially Fe, Al, and Ca, although notable levels of Na and Mg were also detected. These species are known to cause numerous operational issues within both power and capture plants, including contributing to deposition in terms of slagging and fouling within the boiler, as well as initiating and catalyzing oxidative degradation of CO_2_ capture solvents. The high levels of emissions detected with the ICP for these species in the coal samples are due to their high concentrations found in the initial fuel. It is likely that the long-term negative impacts caused by such species are likely to be greater for the utilization of this coal in comparison to this biomass–increased levels of solvent degradation can lead to significantly greater costs for capture.

The fuel analysis for the biomass revealed that this is in reality quite a ‘clean’ fuel. The levels of contamination with many of the transition/heavy metals are quite low and it is only K, Mg, and Ca that are found in concentrations that are noticeably greater than that of the coal feedstock. Even the sulfur and chlorine concentrations were minimal. This clean biomass fuel therefore appeared to perform considerably better in terms of metal emissions than a coal that is typically used for energy generation in thermal power plants in the UK. Apart from K, and some of the Zn readings, metal aerosol release from the combustion of this biomass was significantly reduced compared to coal under the same operating conditions. This is promising for the deployment of dedicated biomass combustion facilities in general and for BECCS systems in particular.

This brings into question what the impact of other biomass fuels would be. As noted in Section 1.2, both alkali metals (which cause deposition) and acidic species (that corrode under the deposits) are generally more prevalent in herbaceous biomass types, such as energy crops and grasses. Potentially, these could have a greater impact on the operability of the furnace and heat recovery components. Such elements, as well as Cr, Cu, Fe, Ni, and V, may also be more predominant in lower-grade and waste fuels, especially energy crops grown on contaminated land for remediation [42]. Subsequently, these may have a much more significant impact on post-combustion capture, especially when using solvent-based systems, with oxidative degradation occurring more rapidly in their presence. Furthermore, the contamination of the high-purity CO_2_ stream with any of these species could be significantly problematic in terms of changing the properties of the supercritical fluid sent for compression, transport and storage.

Changing the fuel feedstocks into such processes has been demonstrated to significantly alter aerosol release profiles. In addition to post-combustion capture strategies, oxy-fuel technologies can also be used to minimize CO_2_ emissions. However, changing the oxidizing environment (from air to an O_2_–CO_2_ mix) could also be an additional factor in determining the release and partitioning of key elements. Oxy-fuel conditions can alter the vaporization of particular species, especially those that are already quite volatile and thus a range of different fuels and combustion environments need to be further explored.

## 5. Conclusions

The prime focus of carbon capture research has so far been on decarbonizing fossil-based power production. BECCS however has the potential for wide-scale deployment and can be a vital technology for meeting emission targets through net negative emissions. Herein, entrained aerosols of alkali and transition metals along with other trace species were compared for coal and biomass combustion via online ICP.

Air-fired combustion trials of a bituminous El Cerrejon coal and Grade A white wood were monitored, with extensive data collected for real-time metal aerosol release. The information revealed that the clean biomass fuel generated significantly lower levels of most metals in aerosol form compared to the coal, and of the various species detected, it was only potassium that had higher aerosol concentrations. Average K emissions generated from biomass combustion were ~6.5 times greater than those for the coal, with peaks of up to 10 times more. This may have a considerable impact on slagging/fouling within the boiler. Na emissions however, which can also contribute to deposition, were notably lower for the biomass. The coal flue gas samples had high concentrations of Fe, Al, and Ca which can further aid deposition, as well as causing issues within the CO_2_ capture plant, specifically oxidative degradation of capture solvents. Coal combustion also generated greater levels of Na and Mg aerosols than the wood.

With the exception of Fe, transition and heavy metal aerosol release was minimal from both fuels. Hg was not found in the biomass flue gas samples at all and was minimal for the coal. This highlights the fact that clean biomass fuels can be used for dedicated power and/or BECCS applications with potentially lower impacts on plant operations, expect for some additional deposition, as is usually expected with biomass. For both fuels, emissions of toxic/heavy metals were minimal, absent or below the detection limit; Cu, As, and Cd were not detected as aerosols for any of the tests.

As demonstrated herein, continuous online detection of metal aerosol emissions can be successfully achieved with the use of ICP-OES. This can aid in the determination of the fate of different species and the partitioning of elements. Such information can help define the mitigation strategies/technologies required to deal with such emissions and this will enable the removal of the technical barriers that face the large-scale deployment of dedicated biomass systems—and BECCS—to achieve lower and potentially net negative carbon emissions. This will develop fundamental knowledge concerning these fuels, which can aid the deployment of BECCS.

## Figures and Tables

**Table 1:** Proximate, ultimate, and elemental analyses of the coal and biomass fuels, assessed via standard methods.

Analysis	Component	El Cerrejon Coal	White Wood
-	As received moisture (wt.%)	7.6	6.7
Proximate analysis (wt.%, dry)	Ash content	3.1	0.7
	Volatile matter	38.4	83.7
	Fixed carbon	58.4	15.6
Ultimate analysis (wt.%, ar)	Carbon	69.44	48.44
	Hydrogen	4.55	6.34
	Nitrogen	1.32	0.15
	Sulphur	0.07	<0.02
	Chlorine	0.03	<0.01
	Oxygen	15.15	37.69
Element oxides in ash (%)	Aluminum, as Al_2_O_3_	16.6	1.9
	Iron, as Fe_2_O_3_	10.8	1.3
	Calcium, as CaO	14.4	27.0
	Magnesium, as MgO	1.9	5.5
	Sodium, as Na_2_O	1.9	1.3
	Potassium, as K_2_O	1.6	10.1
	Silicon, as SiO_2_	39.9	13.6
Trace metals (mg/kg fuel)	Arsenic	1.3	0.3
	Cadmium	0.2	0.1
	Chromium	0.3	2.2
	Copper	3.2	2.6
	Nickel	3.6	0.7
	Mercury	<0.1	<0.1
	Vanadium	11.6	<0.6
	Zinc	4.5	10.2

**Table 2:** Operating conditions for the CTF during the coal and biomass firing tests.

Operating Parameter	Coal-Firing	Biomass-Firing
Fuel net calorific value (MJ/kg)	28.4	18.1
Fuel mass flow (kg/h)	25.3	39.8
Net thermal input (kW)	200	200
Furnace exit O_2_ (vol%, dry)	4.0	3.6

**Table 3:** Operating parameters used for the Spectro CIROS^CCD^ inductively coupled plasma optical emission spectrometer (ICP-OES) generator and nebulizer.

ICP Operating Parameter	Value
Generator frequency (MHz)	27.15
Plasma power (W)	1700
Coolant/plasma gas flow (L/min)	20
Auxiliary gas flow (L/min)	1.0
Nebulizer/carrier gas flow (L/min)	0.6
Sample flow from peristaltic pump (L/min)	0.2
Nebulizer temperature at desolvator (°C)	150
Nebulizer temperature at condenser (°C)	2

**Table 4:** Emissions spectra (wavelengths) and detection limits for the elements assessed via ICP.

Element	Spectral Line	Detection Limit (mg/m^3^)	
		Lower	Upper
Al	396.152	0.04810	86.9
As	189.042	0.24500	86.9
Ca	422.673	0.05000	86.9
Cd	228.802	0.03400	86.9
Cr	267.716	0.03540	86.9
Cu	324.754	0.00339	86.9
Fe	259.941	0.00339	86.9
Hg	253.652	0.00920	27.1
K	766.491	0.04200	86.9
Mg	279.553	0.03520	86.9
Na	589.592	0.06890	86.9
Ni	231.604	0.00917	86.9
V	292.464	0.04670	86.9
Zn	213.856	0.02620	86.9

**Table 5:** Relative emission concentration ratios from coal and biomass combustion, compared to the data for the coal averages of the respective species.

Metal Emissions		El Cerrejon Coal		White Wood	
		Average	Maximum	Average	Maximum
Alkali and alkali earth metals	Potassium (K 766.491)	1	1.80	6.53	10.41
	Sodium (Na 589.592)	1	1.59	0.19	0.37
	Magnesium (Mg 279.553)	1	1.70	0.18	0.40
	Calcium (Ca 422.673)	1	2.05	0.29	0.70
Transition metals	Iron (Fe 259.941)	1	1.72	0.04	0.07
	Nickel (Ni 231.604)	1	1.72	0.68	1.04
	Vanadium (V 292.464)	1	2.03	0.03	0.05
	Zinc (Zn 213.856)	1	2.04	1.13	1.84
Heavy/toxic and other metals	Aluminum (Al 396.152)	1	1.84	0.02	0.04
	Mercury (Hg 253.652)	1	1.71	0.00	0.00
	Chromium (Cr 267.716)	1	1.63	1.00	1.53

## Nomenclature and Abbreviations

BECCS	bioenergy with carbon capture and storage
CCS	carbon capture and storage
CIROS^CCD^	CIRcular Optical System with Charge Coupled Device
CTF	combustion test facility
ICP-OES	inductively coupled plasma optical emission spectrometer
PACT	Pilot-scale Advanced CO_2_ Capture Technology
